# Uncertainty as a Gateway to Beauty: The Impact of Uncertainty Reduction on Art Appreciation

**DOI:** 10.3390/bs16020286

**Published:** 2026-02-16

**Authors:** Yan Duan, Yonghui Hou, Tingting Ouyang, Wanyi Chen, Chenjing Wu, Wei Zhang, Xianyou He

**Affiliations:** 1 Center for Studies of Psychological Application, South China Normal University, Guangzhou 510631, China; 2022010243@m.scnu.edu.cn (Y.D.); 2023023858@m.scnu.edu.cn (Y.H.); 2023023829@m.scnu.edu.cn (T.O.); wuchenjing@m.scnu.edu.cn (C.W.); cheungwai@m.scnu.edu.cn (W.Z.); 2 Key Laboratory of Brain, Cognition and Education Sciences (South China Normal University), Ministry of Education, Guangzhou 510631, China; 3 School of Psychology, South China Normal University, Guangzhou 510631, China; psychenwy@m.scnu.edu.cn; 4 Guangdong Key Laboratory of Mental Health and Cognitive Science, South China Normal University, Guangzhou 510631, China; 5 Philosophy and Social Science Laboratory of Reading and Development in Children and Adolescents (South China Normal University), Ministry of Education, Guangzhou 510631, China

**Keywords:** art appreciation, uncertainty, meaning-making, Aesthetic of Reception theory

## Abstract

Art theory suggests that the pleasure of art appreciation stems from resolving uncertainty, yet empirical support for this idea remains limited. To address this gap, we conducted three studies (*N* = 1127), providing empirical evidence for the Aesthetic of Reception theory. Our findings reveal that reducing uncertainty enhances art appreciation and alleviates boredom, with meaning-making being the most effective strategy (Study 1). Specifically, meaning-making alignment with the artist’s intent leads to more favorable judgments for artworks with low-to-moderate uncertainty. Conversely, highly uncertain paintings are more appreciated when viewers create self-relevant narratives (Study 2). Furthermore, the relationship between uncertainty reduction and aesthetic experience is mediated by the satisfaction of certainty needs (Study 3). These findings suggest that viewers should actively and creatively fill the uncertainty in artworks through meaning-making, fulfilling their need for certainty and transforming uncertainty to a rewarding aesthetic experience while reducing boredom. Overall, our research validates the Aesthetic of Reception theory and offers valuable insights for aesthetic education, encouraging deeper engagement with uncertain artworks.

## 1. Introduction

The secret of being a bore is to tell everything.

—Voltaire

A fundamental principle of human cognition is the drive to minimize uncertainty. In our daily lives, uncertainty represents a gap in our understanding, signaling a potential loss of control and prompting feelings of anxiety (Hirsh et al., 2012; Proulx et al., 2012). Consequently, we are powerfully motivated to resolve ambiguities and maintain a coherent, predictable model of our world. This aversion, however, stands in stark contrast to our engagement with the arts. From enigmatic literature to abstract painting, we often seek out and cherish works characterized by ambiguity and indeterminacy (Jakesch & Leder, 2009). This creates a fascinating paradox: Why do we find pleasure in an experience that, in most other contexts, we strive to avoid? We propose that the key lies not in uncertainty per se but in the rewarding process of transforming uncertainty to meaning via active engagement.

The relationship between uncertainty and aesthetic pleasure has been a central, yet contentious, topic in empirical aesthetics. One influential line of thought—rooted in processing fluency theory and predictive processing frameworks—suggests that pleasure arises from cognitive ease and successful prediction. Predictive coding theory describes the brain as an inference engine, continuously generating expectations and minimizing prediction error (Friston, 2010; Reber et al., 2004). A mismatch between prediction and input creates uncertainty, which the brain is motivated to reduce. Successful resolution of this mismatch is intrinsically rewarding, sometimes producing an “Aesthetic Aha!” moment when a hidden order is suddenly perceived (Muth et al., 2013; Van de Cruys & Wagemans, 2011a). Similar effects have been documented across modalities such as in music, where expectation violations enhance pleasure if resolved (Brielmann & Dayan, 2022; Cheung et al., 2019; Sarasso et al., 2020; Thompson et al., 2023). Crucially, recent predictive-processing accounts suggest that aesthetic pleasure is not a simple function of low prediction error but is closely tied to the dynamics of error reduction over time—i.e., the felt sense of making progress in organizing an initially ambiguous input into a meaningful pattern (Frascaroli et al., 2024). In this spirit, work in this tradition highlights that the brain can “reward progress toward organizing the perceptual field into a meaningful configuration” rather than merely the attainment of a static state of certainty. Accordingly, in our model, “successful resolution/engagement” refers to a subjective, process-based increase in interpretability and predictability (experienced clarity) and does not require converging on a single objectively correct interpretation.

However, maximal certainty is rarely optimal. If resolution alone drove pleasure, the most predictable artworks would be the most liked—yet excessive predictability often leads to boredom (Danckert & Elpidorou, 2023). Berlyne’s (1970) arousal theory proposed an inverted-U relationship, where aesthetic pleasure peaks at moderate levels of novelty, complexity, and uncertainty. In line with this, empirical studies show that moderate ambiguity in visual art can enhance curiosity and interest, while too little or too much ambiguity reduces engagement (Jakesch & Leder, 2009; Muth et al., 2015). Later work questioned whether resolution is the main driver. For instance, Muth et al. (2015) found that an artwork’s solvability was not always linked to liking and that the process of elaboration—sustained engagement with ambiguity—can itself be rewarding. They proposed that “solvability” may even negatively correlate with interest in certain cases, suggesting that continuous interpretive engagement might better sustain aesthetic pleasure. This resonates with the “effort after meaning” principle (Bartlett, 1932) and with reception-based theories emphasizing the pleasure of interpretive engagement.

The Aesthetic of Reception theory (Iser, 1978) provides a conceptual bridge between these perspectives. It holds that artworks are inherently indeterminate, containing “gaps” that the audience must actively fill. Aesthetic experience emerges through “concretization,” where the viewer resolves ambiguities, connects disparate elements, and co-creates meaning. The related concept of “aesthetics of indeterminacy” (Ingarden, 1965; Kemp & Meyer, 1985) highlights how artists deliberately leave parts of a work open-ended, thereby inviting active viewer participation. The pleasure is not located solely in initial ambiguity or final certainty but in the dynamic process of moving from one to the other. This aligns with predictive coding’s emphasis on uncertainty reduction while also highlighting the role of active cognitive participation.

By integrating these perspectives, we propose a meaning-driven uncertainty resolution model. In this model, encountering artistic uncertainty acts as a catalyst for “active inference,” where the viewer is not a passive recipient of sensory input but an agent who actively “forages” for meaning to update their internal models (Frascaroli et al., 2024). Encountering artistic uncertainty motivates viewers to engage in meaning-making, which is rewarding because it both reduces prediction error (predictive coding) and satisfies a fundamental need for certainty (Danckert & Elpidorou, 2023; Dewey, 1929). Crucially, the reward stems from active, successful engagement, as emphasized by reception theory and the “effort after meaning” concept. This conceptualization also aligns with theories of intrinsic motivation, where challenges optimally matched to one’s interpretive capacity yield heightened engagement and enjoyment (Csikszentmihalyi, 2000; Silvia, 2008).

To examine these ideas, we distinguish between three modes of engagement with artwork: mere exposure, meaning-viewing, and meaning-making. Mere exposure increases familiarity and fluency, enhancing liking, even for initially challenging art (Belke et al., 2015; Jakesch et al., 2017; Zajonc, 1968). In ambiguous or abstract paintings, repeated exposure can lead to sustained or even increasing positive evaluations, likely due to progressive familiarity with visual features (Belke et al., 2010). Meaning-viewing—receiving interpretive information, such as titles—can provide semantic scaffolding and reduce ambiguity (Leder et al., 2004; Millis, 2001; Pelowski et al., 2017). In contrast, meaning-making requires the viewer to actively generate interpretations, embodying the co-creator role central to reception theory. Although meaning-viewing provides external semantic scaffolding that can reduce ambiguity, we expect active meaning-making to produce the largest decrease in subjective uncertainty. One reason is that self-generated interpretations are often more deeply processed and more firmly integrated into existing cognitive schemas than information received passively (i.e., the generation effect; Slamecka & Graf, 1978). In addition, externally provided expert interpretations may not always match a novice viewer’s personal knowledge or experiential framework; when the explanation feels insufficiently “owned” by the viewer, a residual sense of uncertainty about understanding can remain. By contrast, meaning-making yields an interpretation constructed within the viewer’s own cognitive framework, thereby increasing subjective fit and strengthening the felt sense of resolution.

Guided by the meaning-driven uncertainty resolution framework, we conceptually distinguish the mode of engagement (the experimental manipulation) from perceived uncertainty reduction (the underlying psychological mechanism). This distinction aligns with the prospect that art serves as a controlled environment for exercising our capacity to manage uncertainty, where the reward is contingent on the transition from ambiguity to coherence (Frascaroli et al., 2024). Given that meaning-making involves deeper interpretive activity and stronger cognitive investment, we expect it to exert the strongest effect on aesthetic responses. These differences motivate the following hypothesis:

**H1.**  *Active meaning-making will yield greater aesthetic benefits than meaning-viewing or mere exposure.*

Building on the meaning-driven uncertainty resolution model, we propose that perceived reduction in uncertainty serves as the key psychological mechanism linking the engagement mode to the aesthetic response. Active meaning-making is expected to produce the largest decrease in subjective uncertainty, which, in turn, drives enhanced aesthetic experience. In the present research, we operationalize “aesthetic benefits” and “enhanced aesthetic experience” as increases in liking, beauty, and pleasure, along with reductions in boredom. This leads to our mediation hypothesis:

**H2.**  *The positive effect of active meaning-making on the aesthetic experience will be mediated by the perceived uncertainty reduction.*

Meaning-making itself varies in source. The “intentional fallacy” debate in art theory distinguishes between pursuing the artist’s intention and constructing personal, self-relevant meaning (Hirsch, 1960; Wimsatt & Beardsley, 1946). Some perspectives argue that authorial intent provides an anchor that can guide interpretation and facilitate perceptual closure (Hirsch, 1960), while others contend that meaning emerges independently of the artist’s original aims (Kjørup, 1971). Some abstract artists reject a single objective meaning (Crowley, 1958; Young, 1997), while others see art as a channel for sharing the artist’s communicative intent (Gao, 2019). Vessel et al. (2021) suggest that both self-association and intent-based approaches can be rewarding, but their effectiveness may depend on the work’s uncertainty level.

**H3.**  *For low- to moderate-uncertainty works, intent-based meaning-making will be more effective; for high-uncertainty works, self-associative meaning-making will be more effective.*

Finally, we examine mechanisms. Navigating ambiguity in art may fulfill the need for certainty, a deep-seated motivation to establish coherence and predictability (Danckert & Elpidorou, 2023; Dewey, 1929). This satisfaction links cognitive engagement to affective reward. Empirical work on need satisfaction in aesthetic contexts suggests that fulfilling epistemic needs can enhance both hedonic and meaningful aspects of art experience (Menninghaus et al., 2019; Schindler et al., 2017).

**H4.**  *Satisfaction of the need for certainty mediates the positive relationship between uncertainty reduction and aesthetic experience.*

Moreover, engagement with uncertain art may mitigate boredom, conceptualized as a crisis of meaning, by promoting interpretive activity and self-relevant reflection (Menninghaus et al., 2017). During periods of monotony, such as during the COVID-19 pandemic, individuals turned to art to restore cognitive engagement and alleviate stagnation (Drake et al., 2024).

**H5.**  *Meaning-making reduces boredom by increasing perceived meaning.*

By testing these hypotheses, which were formulated prior to data collection based on our theoretical framework, our research addresses fundamental questions about how people transform uncertainty to aesthetic value. The meaning-driven uncertainty resolution model integrates predictive processing, arousal, and reception-based theories, providing a comprehensive framework for understanding why ambiguity can be pleasurable and how active engagement amplifies aesthetic experience. Findings from this work have implications not only for psychological theories of art but also for educational practice, public engagement with challenging artworks, and therapeutic interventions that harness the cognitive and emotional benefits of meaning-making.

## 2. Study 1

Study 1 provides an initial test of the engagement-mode account by comparing four approaches to uncertainty resolution (meaning-making, meaning-viewing, mere exposure, and control). This study primarily tests whether meaning-making yields greater aesthetic gains than alternative modes (H1) and whether these effects are mediated by perceived uncertainty reduction (H2). We also examine boredom as an additional outcome relevant to our broader theorizing about meaning-based engagement; reductions in boredom would be consistent with our broader predictions regarding meaning-making (H5).

### 2.1. Method (Study 1)

#### 2.1.1. Study 1 Participants

Acknowledging that G*Power (version 3.1.9.7; Heinrich Heine University Düsseldorf, Düsseldorf, Germany; Faul et al., 2007) does not directly compute power for linear mixed-effect models, we used it to obtain a conservative estimate for a conventional 3 (painting type) × 4 (reduction mode) between-subject ANOVA. This analysis indicated that a minimum of 225 participants would be required to detect a medium-sized interaction effect (*f* = 0.25) with 80% power at α = 0.05. To ensure high statistical power and the robustness of our findings, we made the decision to substantially over-recruit, resulting in our final sample of 519 participants (230 men and 289 women; *M*_age_ = 22.50, *SD* = 3.29). This large sample provides excellent power to detect the effects of interest, even within our complex 12-group design. Study 1 employed a mixed recruitment strategy (offline classroom recruitment and online participation) over a relatively extended period, which yielded a more gender-balanced sample. Participants were screened via self-report and excluded if they had majored in fine arts or received extended formal training in art-related disciplines (e.g., structured training in painting, sculpture, or art theory).

#### 2.1.2. Materials (Study 1)

We conducted a pre-experiment to establish different levels of uncertainty in Western paintings. Initially, we selected 54 Western paintings from publicly accessible art websites, representing diverse art historical periods, genres, styles, artists, and levels of abstraction. These paintings were randomized and divided into three groups of approximately seventeen artworks each for experimental measurements.

One hundred and one participants (twenty-three men, *M_age_* = 20.92, *SD* = 2.42), who did not participate in the main experiment, were randomly assigned to three groups to rate these paintings for perceived uncertainty, beauty, liking, and familiarity. Perceived uncertainty was measured using the following question: “To what extent do you perceive uncertainty in this painting?” All the ratings used a seven-point scale (1 = *not at all*, 7 = *very much*). Additionally, we assessed the emotional valence of each artwork on a seven-point scale (1 = *very negative*, 7 = *very positive*). Ultimately, we selected eight paintings for each category: low uncertainty (*M* = 2.01, *SD* = 0.27), moderate uncertainty (*M* = 3.72, *SD* = 0.47), and high uncertainty (*M* = 6.02, *SD* = 0.31). One-way ANOVA confirmed significant differences in perceived uncertainty across the three final stimulus sets: *F*(2, 21) = 250.56, *p* < 0.001. In addition to uncertainty, baseline beauty, liking, and familiarity differed across the three stimulus groups (*p*s < 0.001). These differences are consistent with established associations between processing difficulty and aesthetic evaluation.

Detailed stimulus information and item-level descriptive statistics are provided in Supplementary Tables S1 and S2. All the paintings were standardized to a height of 847 pixels while maintaining their original aspect ratios. The exact pixel dimensions of each stimulus are provided in Supplementary Table S1.

#### 2.1.3. Study 1 Procedure

The study employed a 3 (painting type: low vs. moderate vs. high) × 4 (uncertainty reduction mode: making meaning vs. viewing meaning vs. mere exposure vs. control) between-subject design. With a total of five hundred nineteen participants randomly assigned to one of the twelve conditions, the average number of participants per condition was approximately forty-three. The procedure consisted of three main steps:

**Pre-test.** Participants were first introduced to the concept of uncertainty in paintings. Specifically, they were informed that uncertainty refers to the presence of ambiguity, openness, and multiple interpretations within an artwork—reflecting how much of the work’s meaning is left unspecified or ‘blank’ by the artist. To ensure a consistent understanding, participants were shown one highly representational painting and one highly abstract painting as examples of low and high uncertainties, respectively. They then viewed eight artworks, rating each on perceived uncertainty, liking, beauty, boredom, and pleasure. Perceived uncertainty was measured with the item: “How uncertain or ambiguous do you find this painting?” All the ratings were made on a seven-point scale (1 = *not at all*, 7 = *very much*).

**Uncertainty Reduction.**

Following the pre-test, participants were randomly assigned to one of four experimental conditions. The eight artworks were presented in a randomized order. Presentation durations were systematically varied across conditions to target different psychological processes (i.e., rapid perceptual fluency versus slower cognitive elaboration).

In the making meaning condition, participants were instructed to actively construct interpretations of each artwork. They were prompted with questions such as, “What meaning or significance do you think this painting conveys?” and “What kind of story does it tell?” Participants had up to 3 min per artwork to type their interpretations into a text box.

In the viewing meaning condition, participants viewed each artwork, accompanied by a brief, expert-generated interpretation displayed on the same screen. They were asked to read the interpretation carefully. Interpretations were standardized in length (approximately 30–50 words in English translation) and provided (a) brief references to salient formal/compositional features (e.g., color, line, and focal regions) and (b) a concise thematic or narrative reading intended to support interpretability for novice viewers. As in the making meaning condition, this task required semantic processing and allowed up to 3 min per artwork.

The mere exposure condition was designed to isolate the effects of bottom-up perceptual fluency, independent of top-down meaning processing. Framed as a memory task, each artwork was presented nine times for 1 s each, with a 1 s fixation cross between presentations. This rapid, repetitive exposure followed the classic paradigm used in mere exposure research (Mrkva & Van Boven, 2020).

In the control condition, participants were given the same total exposure time and comparable cognitive effort as the elaborative tasks. They viewed each artwork for up to 3 min while typing their opinions on an unrelated topic (e.g., “childhood moral development”).

The differences in exposure duration across conditions were inherent to the intended manipulation of the cognitive engagement, with elaborative tasks requiring extended viewing time and the mere exposure condition designed to capture brief, repeated perceptual processing.

Finally, participants completed a post-test on the same eight artworks, using identical items to those in the pre-test. The order of the artworks was randomized.

### 2.2. Data Analyses

We employed a pre–post experimental design to test the effects of uncertainty reduction. Change scores were calculated by subtracting pre-test ratings from post-test ratings, resulting in difference scores for perceived uncertainty, liking, beauty, boredom, and pleasure. For the sake of clarity in interpreting the complex interactions in our design, we opted for this approach to directly quantify the magnitude of the change following our interventions. We present the change score analysis herein, focusing on the clear main and interaction effects of our experimental manipulations on the magnitude of the change. Additionally, we took the reverse of the perceived uncertainty and boredom difference scores to represent the degrees of uncertainty reduction and boredom reduction, respectively.

Linear mixed-effect analyses were conducted using the lme4 package (Bates et al., 2015) in R (version 4.2.2; R Foundation for Statistical Computing, Vienna, Austria) to assess whether perceived uncertainty reduction influences aesthetic judgments, particularly painting beauty. The *p*-values for parameter estimates were derived using Satterthwaite’s approximation, which was implemented in the lmerTest package (Kuznetsova et al., 2017).

For each dependent variable, we constructed a model with random intercepts for both participants and items (artworks). This approach statistically controls for baseline individual differences in rating styles among participants and inherent differences in appeal among the artworks. Because the condition was manipulated between participants, by-participant random slopes for the condition are not identifiable. We evaluated more complex random-effect structures for within-subject factors and item-level variability; however, these models often produced convergence warnings and/or singular fits, consistent with overparameterization given the available repeated observations. We, therefore, adopted a parsimonious random-intercept structure to ensure stable estimation (Bates et al., 2015; Matuschek et al., 2017). Fixed effects in our models included the painting type, uncertainty reduction mode, and their interaction.

Regression coefficients (*b*), standard errors (*SE*s), and *t*-values are reported for each model. For all the post hoc pairwise comparisons of the estimated marginal means, *p*-values were adjusted using the Tukey method to correct for multiple comparisons. Visualizations were generated with the effect package (Fox & Weisberg, 2018). In addition to *p*-values, we report marginal and conditional *R*^2^ values for mixed-effect models to indicate the variance explained by fixed effects alone and by the full model (fixed + random effects).

It is important to note our reporting convention: The linear mixed-effect model with both fixed and random effects, including regression coefficients relative to the specified reference group, is presented in the tables. In the main text, to directly test our hypotheses, we primarily report the results of specific post hoc pairwise contrasts. Consequently, the numerical values for coefficients in the text (from pairwise contrasts) may differ from those in the tables (from the model summary).

### 2.3. Results for Study 1

#### 2.3.1. Manipulation Check (Study 1)

To verify the effectiveness of our uncertainty categorization (low, moderate, and high), we analyzed participants’ baseline (pre-manipulation) ratings of perceived uncertainty collected at the beginning of Study 1. Participants who viewed low-uncertainty paintings reported significantly lower uncertainty levels (*M* = 2.66, *SD* = 0.72) compared to those who viewed moderate- (*M* = 4.70, *SD* = 0.97) and high-uncertainty paintings (*M* = 5.52, *SD* = 0.93), *F*(2, 507) = 482.56, *p* < 0.001, and $\eta_{p}^{2}$ = 0.65. These results robustly confirm the efficacy of our uncertainty manipulation.

#### 2.3.2. Effects of Uncertainty Reduction Modes on Uncertainty and Aesthetic Experience

One of our main goals was to test how different modes of uncertainty reduction affected the aesthetic experience of paintings. A linear mixed model was used to analyze this effect. Uncertainty reduction, increased liking, beauty, pleasure, and boredom reduction were used as dependent variables. Painting types and uncertainty reduction modes were used as fixed factors, and painting stimuli and participants were used as random intercepts. The “making meaning” condition was set as the reference level in all the mixed-effect models because it served as the theoretically central comparison.

**Uncertainty Reduction.**

**Figure 1:**
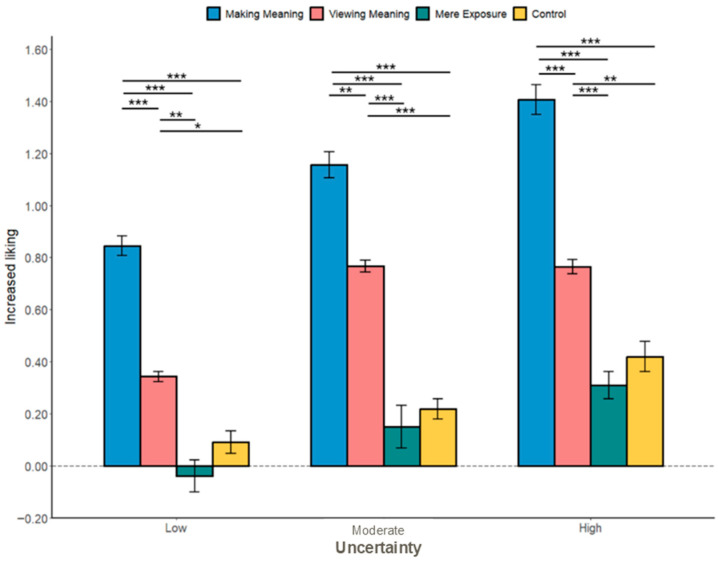
The effect of the uncertainty reduction mode on uncertainty reduction across uncertainty types for Study 1. *** *p* < 0.001, ** *p* < 0.01, and * *p* < 0.05. Error bars represent the standard error of the mean calculated across all the individual trials.

The results indicated that the “making meaning” mode led to a greater reduction in uncertainty compared to the “viewing meaning” mode (*b* = 0.37, *SE* = 0.08, *t*(507) = 4.76, *p* < 0.001), mere exposure (*b* = 0.52, *SE* = 0.08, *t*(507) = 6.84, *p* < 0.001), and control group (*b* = 0.67, *SE* = 0.08, *t*(507) = 8.91, *p* < 0.001; see Figure 1, Table 1 and Supplementary Table S3 for the remaining fixed effects and means).

More specifically, a significant interaction effect showed that for low-uncertainty paintings, there was no significant difference between “making meaning” and other modes (viewing meaning: *b* = −0.13, *SE* = 0.13, *t*(507) = −0.94, and *p* = 0.349; mere exposure: *b* = −0.13, *SE* = 0.13, *t*(507) = −0.96, and *p* = 0.338, and the control group: *b* = 0.12, *SE* = 0.13, *t*(507) = 0.99, and *p* = 0.322).

Moreover, for moderate-uncertainty paintings, “making meaning” decreased more uncertainty than mere exposure and the control group (mere exposure: *b* = 0.42, *SE* = 0.14, *t*(507) = 3.09, and *p* = 0.002; the control group: *b* = 0.72, *SE* = 0.13, *t*(507) = 5.55, and *p* < 0.001) but no difference with “viewing meaning” (viewing meaning: *b* = 0.21, *SE* = 0.13, *t*(507) = 1.66, and *p* = 0.098).

For high-uncertainty paintings, “making meaning” was more effective in reducing uncertainty compared to other modes (viewing meaning: *b* = 1.00, *SE* = 0.13, *t*(507) = 7.55, and *p* < 0.001; mere exposure: *b* = 1.26, *SE* = 0.12, *t*(507) = 10.19, and *p* < 0.001, and the control group: *b* = 1.17, *SE* = 0.13, *t*(507) = 8.84, and *p* < 0.001).

**Increased Liking.**

**Figure 2:**
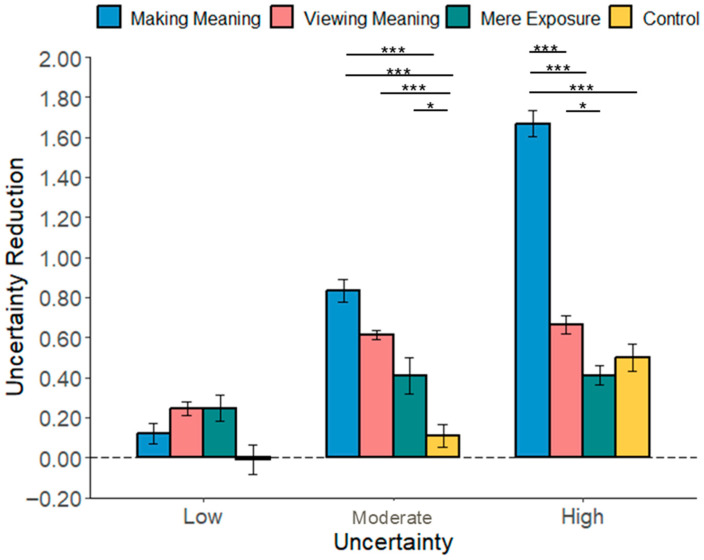
The effect of the engagement mode on uncertainty reduction across baseline uncertainty levels (Study 1). *** *p* < 0.001 and * *p* < 0.05. Error bars represent the standard error of the mean calculated across all the individual trials.

The results showed that the “making meaning” mode led to greater increases in liking than the “viewing meaning” mode (*b* = 0.51, *SE* = 0.06, *t*(507) = 8.72, and *p* < 0.001), the “mere exposure” (*b* = 1.00, *SE* = 0.06, *t*(507) = 17.21, and *p* < 0.001), and the control group (*b* = 0.89, *SE* = 0.06, *t*(507) = 15.45, and *p* < 0.001). There was no significant interaction between uncertainty reduction modes and picture types, *p*s > 0.05 (see Figure 2).

**Increased Beauty.**

The “making meaning” mode also produced higher beauty ratings than the “viewing meaning” mode (*b* = 0.47, *SE* = 0.06, *t*(507) = 8.53, and *p* < 0.001), mere exposure (*b* = 0.90, *SE* = 0.05, *t*(507) = 16.63, and *p* < 0.001), and the control group (*b* = 0.86, *SE* = 0.05, *t*(507) = 15.73, and *p* < 0.001). A significant interaction between painting types and condition emerged for increased beauty, *χ*^2^(6) = 14.95, *p* = 0.021, indicating that the effectiveness of uncertainty reduction modes differed across levels of uncertainty. Specifically, the contrast between high-uncertainty paintings in the control condition differed from those in the reference condition (see Table 1).

**Boredom Reduction.**

Our results indicated that “making meaning” significantly reduced boredom compared to “viewing meaning” (*b* = 0.16, *SE* = 0.08, *t*(507) = 2.11, and *p* = 0.036), mere exposure (*b* = 0.41, *SE* = 0.07, *t*(507) = 5.48, and *p* < 0.001), and the control group (*b* = 0.60, *SE* = 0.07, *t*(507) = 8.02, and *p* < 0.001).

The analysis revealed a significant interaction effect between painting types and uncertainty reduction modes. For low-uncertainty paintings, making meaning decreased boredom significantly more than the control group (*b* = 0.28, *SE* = 0.13, *t*(507) = 2.20, and *p* = 0.028) but did not differ significantly from “viewing meaning” (*b* = −0.01, *SE* = 0.13, *t*(507) = −0.07, and *p* = 0.943) or mere exposure (*b* = 0.23, *SE* = 0.13, *t*(507) = 1.80, and *p* = 0.072).

For moderate-uncertainty paintings, “making meaning” showed greater boredom reduction than the control group (*b* = 0.58, *SE* = 0.13, *t*(507) = 4.52, and *p* < 0.001). There was no significant difference between making meaning and viewing meaning (b = −0.09, *SE* = 0.13, *t*(507) = −0.71, and *p* = 0.480) and mere exposure (*b* = 0.09, *SE* = 0.13, *t*(507) = 0.68, and *p* = 0.496).

For high-uncertainty paintings, “making meaning” decreased boredom significantly more than “viewing meaning” (*b* = 0.58, *SE* = 0.13, *t*(507) = 4.42, and *p* < 0.001), mere exposure (*b* = 0.90, *SE* = 0.12, *t*(507) = 7.39, and *p* < 0.001), and the control group (*b* = 0.93, *SE* = 0.12, *t*(507) = 7.13, and *p* < 0.001).

**Increased Pleasure.**

The results showed that “making meaning” significantly increased pleasure more than the other modes: “viewing meaning” (*b* = 0.46, *SE* = 0.06, *t*(507) = 8.02, and *p* < 0.001; mere exposure: *b* = 0.86, *SE* = 0.06, *t*(507) = 15.17, and *p* < 0.001, and the control group: *b* = 0.88, *SE* = 0.06, *t*(507) = 15.17, and *p* < 0.001). The interaction between painting types and the uncertainty reduction mode was not significant for increased pleasure (*p*s > 0.05).

These findings suggest that making meaning is the most effective mode in aesthetic experience and decreasing boredom compared to the other modes, particularly for paintings with moderate and higher levels of uncertainty.

#### 2.3.3. The Mediating Role of Perceived Uncertainty Reduction

Having established a causal link between the reduction modes and aesthetic experience, we next sought to examine the psychological mechanism underlying this effect. We hypothesized that the effect of the experimental conditions on the aesthetic experience is mediated by the perceived reduction in uncertainty.

**Figure 3:**
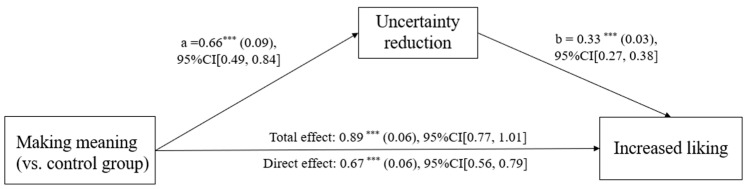
Uncertainty reduction mediated the effect of making meaning on increased liking in Study 1. *** *p* < 0.001.

To test this, we conducted a mediation analysis using the bruceR package (version 2.1.0; Bao, 2023; Shanghai, China) for R (version 4.2.2; R Core Team, Vienna, Austria), employing Model 4, 5000 bootstrapping samples (Hayes & Scharkow, 2013) to explore the mediating role of uncertainty reduction. We conducted the uncertainty reduction modes as the independent variable, uncertainty reduction as the mediator, and increased liking as the dependent variable. We ran a mediation analysis comparing the making meaning condition (coded as “1”) to the control condition (coded as “0”). The results showed that uncertainty reduction significantly mediated the relationship between “making meaning” and increased liking, *b* = 0.22, *SE* = 0.04, 95% CI [0.14, 0.30] (see Figure 3).

A similar pattern was observed for increased beauty. The mediation analysis revealed a significant indirect effect of the “making meaning” mode (vs. control) through uncertainty reduction (*b* = 0.07, *SE* = 0.01, 95% CI [0.05, 0.10]). The direct effect of the condition on beauty remained significant (*b* = 0.15, *SE* = 0.02, *t* = 7.81, and *p* < 0.001), indicating partial mediation. Together, these findings suggest that active meaning-making enhances the aesthetic experience, in part, by facilitating reductions in subjective uncertainty.

### 2.4. Discussion (Study 1)

These findings highlight that “making meaning” is the most effective approach for enhancing aesthetic experiences and reducing boredom, especially with artworks exhibiting moderate- to high-uncertainty levels. Consistent with our hypothesis, reducing uncertainty enhances enjoyment, with “making meaning” outperforming the other modes in fostering viewers’ engagement and pleasure. Mediation analysis further suggests that the aesthetic advantages of “making meaning” are facilitated through the process of uncertainty reduction. Together, these findings provide direct support for Hypotheses 1 and 2, establishing that active meaning-making enhances the aesthetic experience and that this effect operates through reductions in perceived uncertainty.

## 3. Study 2

Study 2 aimed to address two main objectives. First, in Study 1, meaning-making involved both the viewers’ self-involvement and their understanding of the painting’s intended meaning. However, self-involvement alone can enhance positive experiences (Mas-Herrero et al., 2018; Vessel et al., 2023), potentially conflating these effects. Second, Study 2 was inspired by the debate in art theory between intentionalism and anti-intentionalism. Therefore, we distinguished two types of meaning-making: one based on the author’s intention and another on self-association, to assess whether the author’s intent is necessary for aesthetic appreciation (H3). It also continues to investigate the underlying mechanism of certainty-need satisfaction (H4). The study design and hypotheses were pre-registered (https://aspredicted.org/nhgw-783b.pdf; accessed on 17 October 2024).

### 3.1. Method (Study 2)

#### 3.1.1. Participants (Study 2)

The sample size for this study was determined using an a priori power analysis conducted using G*Power (Faul et al., 2007). Following Cohen’s (1988) convention, we aimed to detect a medium-sized interaction effect (*f* = 0.25) for our 3 × 2 between-subject design. The analysis indicated that a minimum sample size of 158 participants was required to achieve 80% power at α = 0.05. To ensure high statistical power, we recruited 239 undergraduate students from a large Chinese public university (61 men and 178 women; *M_age_* = 21.62 years, *SD* = 3.53). The participants were screened via self-report and excluded if they had majored in fine arts or received extended formal training in art-related disciplines (e.g., structured training in painting, sculpture, or art theory).

#### 3.1.2. Study 2 Procedure

This study used a 3 (painting types: high vs. moderate vs. low) × 2 (meaning-making types: author’s intention vs. self-association) between-subject design. With a total of two hundred thirty-nine participants randomly assigned to one of the six conditions, the average number of participants per condition was approximately forty. The artworks were the same as those in Study 1.

In the first phase, the participants in all six conditions completed a pre-test rating of perceived uncertainty and liking for the eight paintings, as in Study 1.

Second, in the “author’s intention” condition, participants tried to speculate the intentions behind the author’s creation and the content being expressed. They were encouraged to imagine the uncertainties the author might have left and then construct meaning based on that. In the “self-association” condition, participants wrote down personal experiences or associations that the image triggered in them and did not need to consider the author’s intention. In the last part, all the participants were involved in the post-test. They also rated their satisfaction of certainty needs for each painting using a single-item scale (i.e., “To what extent did you feel a sense of certainty regarding this painting?”).

### 3.2. Results (Study 2)

To compare the differential effects of two types of meaning-making on aesthetic judgments, we utilized the lme4 package for R (Bates et al., 2015), modeling the effects of meaning-making types and painting types (including interaction terms) on judgments. Random intercepts were specified for participants and painting items (see Table 2 and Supplementary Table S4 for the remaining fixed effects and means). For all the post hoc pairwise comparisons of the estimated marginal means, *p*-values were adjusted using the Tukey method to correct for multiple comparisons.

#### 3.2.1. Type of Meaning-Making and Uncertainty Reduction

We also examined whether the type of meaning-making affected the reduction in uncertainty. The main effect of the meaning-making type was not significant (*b* = 0.17, *SE* = 0.09, *t*(233) = 1.89, and *p* = 0.06).

There was also a significant interaction between meaning-making types and painting types. For low-uncertainty paintings, differences between meaning-making types were insignificant (*b* = 0.29, *SE* = 0.16, *t*(233) = 1.83, and *p* = 0.07). For moderate-uncertainty paintings, meaning-making based on the author’s intention reduced perceived uncertainty more than self-association (*b* = 0.62, *SE* = 0.15, *t*(233) = 4.17, and *p* < 0.001). In contrast, for high-uncertainty paintings, self-association reduced uncertainty more than the author’s intention (*b* = −0.42, *SE* = 0.15, *t*(233) = −2.79, and *p* = 0.006).

#### 3.2.2. Increased Liking

Meaning-making based on the author’s intention increased liking more than self-association (*b* = 0.29, *SE* = 0.09, *t*(233) = 3.02, and *p* = 0.002).

**Figure 4:**
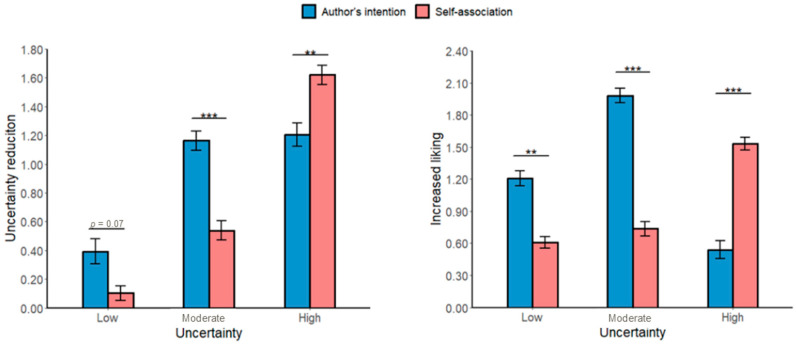
The effects of the type of meaning-making on uncertainty reduction and increased liking across uncertainty types for Study 2. *** *p* < 0.001 ang ** *p* < 0.01. Error bars represent the standard error of the mean calculated across all the individual trials.

A significant interaction between meaning-making types and painting types emerged: For low- and moderate-uncertainty paintings, liking was more significant for the author’s intention than for self-association (low-uncertainty paintings: *b* = 0.60, *SE* = 0.17, *t*(233) = 3.51, and *p* =0.001; moderate-uncertainty paintings: *b* = 1.25, *SE* = 0.16, *t*(233) = 7.78, and *p* < 0.001). However, for high-uncertainty paintings, self-association led to a higher increase in liking than that based on the author’s intention (*b* = −0.99, *SE* = 0.16, *t*(233) = −6.19, and *p* < 0.001; see Figure 4).

#### 3.2.3. Increased Beauty (Study 2)

The author’s intention increased the perceived beauty more than self-association (*b* = 0.27, *SE* = 0.10, *t*(233) = 2.78, and *p* = 0.006). The interaction between meaning-making types and painting types was significant: For low- and moderate-uncertainty paintings, beauty ratings were higher for the author’s intention than for self-association (low-uncertainty paintings: *b* = 0.59, *SE* = 0.18, *t*(233) = 3.30, and *p* = 0.001; moderate-uncertainty paintings: *b* = 1.23, *SE* = 0.17, *t*(233) = 7.37, and *p* < 0.001). For high-uncertainty paintings, self-association increased beauty ratings more than the author’s intention (*b* = −0.10, *SE* = 0.17, *t*(233) = −5.99, and *p* < 0.001).

#### 3.2.4. Boredom Reduction (Study 2)

A significant interaction between the meaning-making and painting types was observed for reduced boredom. For low- and moderate-uncertainty paintings, the author’s intention was more effective in lowering boredom than self-association (low-uncertainty paintings: *b* = 0.35, *SE* = 0.16, *t*(233) = 2.16, and *p* = 0.033; moderate-uncertainty paintings: *b* = 0.36, *SE* = 0.15, *t*(233) = 2.31, and *p* = 0.022). However, for high-uncertainty paintings, self-association reduced boredom more than the author’s intention (*b* = −0.73, *SE* = 0.15, *t*(233) = −4.76, and *p* < 0.001).

#### 3.2.5. Increased Pleasure (Study 2)

Meaning-making based on the author’s intention yielded greater pleasure than self-association (*b* = 0.24, *SE* = 0.10, *t*(233) = 2.25, and *p* = 0.025). A significant interaction indicated that for low- and moderate-uncertainty paintings, the author’s intention produced greater pleasure than self-association (low-uncertainty paintings: *b* = 0.47, *SE* = 0.19, *t*(233) = 2.51, and *p* = 0.013; moderate-uncertainty paintings: *b* = 1.14, *SE* = 0.18, *t*(233) = 6.43, and *p* < 0.001). For high-uncertainty paintings, self-association led to higher pleasure than the author’s intention (*b* = −0.91, *SE* = 0.18, *t*(233) = −5.13, and *p* < 0.001).

#### 3.2.6. Mediation via the Satisfaction of Certainty Needs

**Figure 5:**
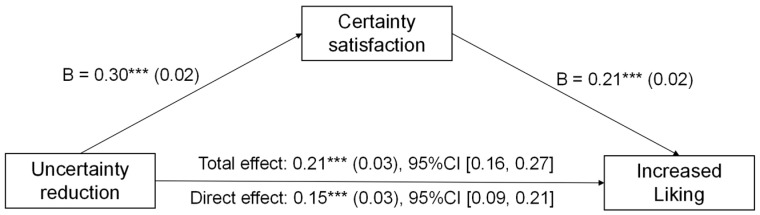
Mediation model for Study 2. *** *p* < 0.001.

We tested whether the certainty-need satisfaction mediated the effect of decreased uncertainty on increased liking using the bruceR package for R. We conducted bootstrapping analyses (Model 4; Hayes & Preacher, 2014; Preacher & Hayes, 2008) with 5000 resamples with 95% CIs for the indirect effects. The results demonstrated a significant indirect effect of uncertainty reduction on heightened liking via the satisfaction of certainty needs (indirect effect = 0.06, 95% CI [0.05, 0.08]; see Figure 5). This underscores the importance of fulfilling certainty needs as a pathway through which reduced uncertainty enhances liking.

### 3.3. Discussion (Study 2)

Study 2’s findings suggest that different types of meaning-making have distinct effects on aesthetic judgments, particularly at varying levels of uncertainty. Meaning-making based on the author’s intention generally led to greater liking, beauty, and pleasure for artworks with low-to-moderate uncertainty. However, self-associative meaning-making proved to be more effective for high-uncertainty paintings, reducing boredom and enhancing enjoyment. This crossover interaction suggests a dynamic shift in the optimal source of meaning. When visual clues are available (low-to-moderate uncertainty), attempting to discern the artist’s intent provides a structured and satisfying “solution” to the visual puzzle. However, when stimuli are highly abstract and external clues are scarce (high uncertainty), the search for a specific authorial intent may become frustrating or futile. In such cases, self-association provides a more accessible and resilient pathway to resolution, as viewers can rely on their own internal experiences to construct valid meanings. The mediation analysis further illustrates that the satisfaction of certainty needs is crucial in transforming reduced uncertainty to increased appreciation, highlighting a meaningful pathway in how certainty fulfillment contributes to positive aesthetic experiences. These findings offer specific support for Hypotheses 3 and 4, demonstrating that the effectiveness of meaning-making strategies depends on the level of uncertainty present in the artwork.

## 4. Study 3

Study 3 sought evidence for the mechanisms underlying the role of uncertainty in aesthetics by experimentally manipulating the mediational process (H4). In this context, we define “certainty satisfaction” not as the belief in a single correct answer but as the confidence that the artwork is meaningful and interpretable. Providing multiple coherent interpretations, therefore, does not necessarily increase ambiguity about correctness but rather strengthens the overall perception of the work as a rich, structured, and meaningful object, thereby satisfying the viewer’s need to find order. We manipulated the certainty satisfaction by presenting participants with paintings with one, three, and five meanings. We predicted that the participants would rate paintings with more meanings as more aesthetically pleasing and that their satisfaction with the need for certainty would be positively correlated with their aesthetic evaluations.

### 4.1. Method for Study 3

#### 4.1.1. Participants for Study 3

An a priori power analysis conducted using G*Power indicated that a sample size of at least 334 participants would be required to achieve a medium effect size (*f* = 0.25; 1-β = 0.95; α = 0.05). We recruited 369 adult Chinese residents (111 men and 258 women; *M*_age_ = 23.68 years, *SD* = 2.77). The participants were screened via self-report and excluded if they had majored in fine arts or received extended formal training in art-related disciplines (e.g., structured training in painting, sculpture, or art theory).

#### 4.1.2. Materials for Study 3

**Painting Materials.**

The painting stimuli used in this study were the same as those in Study 1.

**Meaning Interpretation Materials.**

We invited five art major students (with over five years of art training) to analyze 24 paintings. Each student provided approximately eight interpretations per painting, each around thirty words long. In total, there were 195 unique interpretations for all the paintings.

To control for potential biases in liking and emotional valence toward the interpretations, we conducted a pre-test with 82 undergraduates (*M*_age_ = 20.57 years, *SD* = 1.84; 39 women and 43 men). These participants were randomly assigned to three groups, each assigned to read the interpretations of the paintings without viewing the actual paintings. They rated each interpretation for liking and emotional valence on a seven-point Likert scale (1 = not at all, 7 = very much). We selected, within each painting, the five interpretations which liking and valence ratings were the closest to the overall mean, thereby minimizing the between-painting variance in the affective tone: *M*_liking_ = 4.90, *SD_liking_* = 0.25, *M*_emotion_ = 4.70, and *SD_emotion_* = 0.37. One-way ANOVA confirmed no significant differences in the liking and emotional valence of interpretations across paintings with varying uncertainty levels, *p*s > 0.05.

#### 4.1.3. Study 3 Procedure

This study used a 3 (painting types: high vs. moderate vs. low) × 3 (number of meanings: one vs. three vs. five) between-subject design. With a total of three hundred sixty-nine participants randomly assigned to one of the nine conditions, there were forty-one participants per condition. Each participant completed a pre-test for eight artworks, then viewed interpretations of the paintings and rated their satisfaction of certainty needs. Certainty-need satisfaction was manipulated by varying the number of interpretations provided for each painting (one vs. three vs. five). Interpretations were displayed as a numbered list alongside the artwork. The three- and five-meaning conditions included the same core interpretation as the one-meaning condition, supplemented with additional nonredundant but non-contradictory perspectives (e.g., formal/compositional cues, title-based framing, and thematic/affective readings). Finally, participants re-evaluated the uncertainty, liking, beauty, boredom, and pleasure of each artwork.

### 4.2. Results (Study 3)

#### 4.2.1. Manipulation Check for Study 3

Confirming the success of our certainty satisfaction manipulation, the participants in the five-meaning condition perceived paintings as being more satisfied with the need for certainty than those in the one- and three-meaning conditions, and the participants in the three-meaning condition perceived paintings as providing greater certainty satisfaction than those in the one-meaning condition (*M*_1-meaning_ = 2.54, *SD* = 0.47 vs. *M*_3-meanings_ = 3.52, *SD* = 0.68 vs. *M*_5-meanings_ = 4.29, *SD* = 0.48), *F*(2, 357) = 304.98, and *p* < 0.001.

#### 4.2.2. Number of Meanings and Increased Liking

**Figure 6:**
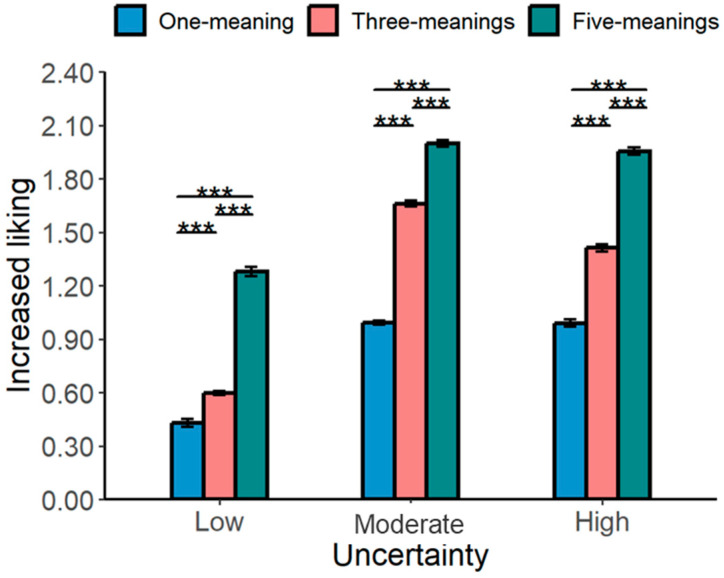
The effect of the number of meanings on increased liking across uncertainty types for Study 3. *** *p* < 0.001. Error bars represent the standard error of the mean calculated across all the individual trials.

A significant main effect of the number of meanings was observed. Paintings with five meanings received higher liking ratings than those with one or three meanings (one meaning vs. five meanings: *b* = −0.94, *SE* = 0.02, *t*(359) = −45.50, and *p* < 0.001; three meanings vs. five meanings: *b* = −0.52, *SE* = 0.02, *t*(359) = −22.13, and *p* < 0.001). Additionally, paintings with one meaning were less liked than those with three meanings (*b* = −0.42, *SE* = 0.02, *t*(359) = −20.88, and *p* < 0.001). A significant interaction between painting types and the number of meanings emerged for increased liking: *χ*^2^(4) = 86.64, *p* < 0.001, indicating that the effect of the meaning quantity depended on the level of uncertainty (see Figure 6, Table 3, and Supplementary Table S5 for the remaining fixed effects and means). All the pairwise post hoc comparisons reported below were computed using emmeans package (version 1.10.1; Iowa City, IA, USA; Lenth, 2024) and adjusted using Tukey’s method for multiple comparisons.

#### 4.2.3. Increased Beauty (Study 3)

A significant main effect of the number of meanings was also observed for beauty ratings. Paintings with more meanings were rated as more beautiful (one meaning vs. three meanings: *b* = −0.41, *SE* = 0.02, *t*(359) = −16.35, and *p* < 0.001; one meaning vs. five meanings: *b* = −0.93, *SE* = 0.02, *t*(359) = −37.45, and *p* < 0.001; three meanings vs. five meanings: *b* = −0.53, *SE* = 0.02, *t*(359) = −21.13, and *p* < 0.001). A significant interaction between the picture type and number of meanings was observed for increased beauty: *χ*^2^(4) = 59.58, *p* < 0.001, suggesting that the impact of additional meanings varied across uncertainty levels.

#### 4.2.4. Boredom Reduction (Study 3)

Our results revealed a significant main effect of the number of meanings. Paintings with more meanings led to lower boredom ratings (one meaning vs. three meanings: *b* = −0.44, *SE* = 0.03, *t*(359) = −14.36, and *p* < 0.001; one meaning vs. three meanings: *b* = −0.77, *SE* = 0.03, *t*(359) = −25.08, and *p* < 0.001; three meanings vs. five meanings: *b* = −0.33, *SE* = 0.03, *t*(359) = −10.75, and *p* < 0.001). A significant interaction between the picture type and number of meanings was found for boredom reduction: *χ*^2^(4) = 44.36, *p* < 0.001.

#### 4.2.5. Increased Pleasure (Study 3)

Similarly, a significant main effect of the number of meanings on pleasure ratings was found, with paintings containing more meanings rated as more pleasurable (one meaning vs. three meanings: *b* = −0.39, *SE* = 0.02, *t*(359) = −16.45, and *p* < 0.001; one meaning vs. three meanings: *b* = −0.93, *SE* = 0.02, *t*(359) = −38.53, and *p* < 0.001; three meanings vs. five meanings: *b* = −0.53, *SE* = 0.02, *t*(359) = −22.11, and *p* < 0.001). A significant interaction between the picture type and number of meanings was also observed for increased pleasure: *χ*^2^(4) = 62.29, *p* < 0.001.

#### 4.2.6. Mediating Effects of Certainty Satisfaction

**Figure 7:**
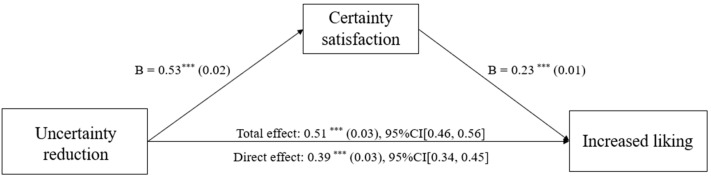
Certainty satisfaction mediated the effect of uncertainty reduction on increased liking in Study 3. *** *p* < 0.001.

We tested whether the satisfaction with the need for certainty mediated the effect of decreased uncertainty on increased liking using the bruceR package for R. We conducted bootstrapping analyses (i.e., Model 4; Hayes & Preacher, 2014; Preacher & Hayes, 2008) with 5000 resamples with 95% CIs for the indirect effects. The results showed that certainty satisfaction significantly indirectly related uncertainty reduction to increased liking (indirect effect: *b* = 0.12, *SE* = 0.01, 95% CI [0.10, 0.14]; see Figure 7). This result verified the indirect relationship between decreased uncertainty and increased liking through certainty satisfaction.

#### 4.2.7. Moderated Mediation

To examine the first-stage- and second-stage-moderated mediation, we applied Model 58 in the process macro (Hayes & Preacher, 2014) with the bruceR package for R. The results showed that the painting type significantly moderated the relationship between the uncertainty reduction and certainty satisfaction, *F*(2, 2938) = 151.00, *p* < 0.001. The indirect effect of the uncertainty reduction on increased liking was the most potent in moderate-uncertainty paintings (*b* = 0.35, *SE* = 0.03, 95% CI [0.2, 0.42]) compared to low-uncertainty (*b* = 0.11, SE = 0.02, 95% CI [0.07, 0.16]) and high-uncertainty paintings (*b* = 0.09, *SE* = 0.01, 95% CI [0.07, 0.12]). These results suggest that fulfilling the need for certainty is particularly influential for moderate-uncertainty artworks.

### 4.3. Discussion (Study 3)

The results from Study 3 support our hypothesis, demonstrating that higher satisfaction of certainty needs was associated with enhanced aesthetic experiences and reduced boredom. Specifically, as the fulfillment of certainty needs increased, participants reported more positive aesthetic judgments, suggesting that the alignment between perceived meanings and personal interpretations plays a crucial role in enhancing enjoyment and reducing feelings of boredom. These findings underscore the essential role of certainty satisfaction in creating enriching and engaging aesthetic experiences. Accordingly, Study 3 provides strong support for Hypothesis 5 by experimentally demonstrating that increasing certainty satisfaction enhances aesthetic evaluation, particularly at varying levels of uncertainty.

## 5. General Discussion

Our findings contribute to understanding a long-standing paradox in psychological aesthetics: Although uncertainty is typically aversive in daily life, it can become a source of pleasure in artistic contexts. We proposed and tested a meaning-driven uncertainty resolution model, which posits that aesthetic pleasure arises not from ambiguity itself but from the viewer’s active, agentic process of resolving it through meaning-making. Across three studies, our findings provide robust support for this model. We demonstrated that while higher uncertainty can initially suppress aesthetic appreciation, this effect is not immutable; it is dynamically modulated by the viewer’s cognitive engagement. Specifically, the act of meaning-making systematically transforms uncertainty from a potential cognitive burden to a source of hedonic and eudaimonic reward, a process mediated by the satisfaction of the fundamental need for certainty.

### 5.1. Theoretical Contributions

Our findings offer a significant contribution by helping to reconcile three competing theoretical stances on uncertainty in aesthetics. First, our results partially support models based on predictive processing and cognitive fluency (Friston, 2010; Reber et al., 2004). The baseline negative correlation between uncertainty and liking confirms that unresolved prediction error is often aversive. However, our subsequent findings challenge a simplistic interpretation of this framework. We demonstrate that the brain is not merely a passive error-reducer; the pathway to error reduction is paramount. This aligns with recent perspectives in predictive processing, which emphasize that the aesthetic value of uncertainty lies in the potential for “learning progress”—the successful and dynamic resolution of prediction errors (Frascaroli et al., 2024; Van de Cruys & Wagemans, 2011a, 2011b). Our results provide empirical evidence that active meaning-making facilitates this dynamic progress more effectively than passive reception.

This leads to our second contribution: a qualification of arousal-based theories, like Berlyne’s (1970), which propose an optimal, moderate level of uncertainty. Our research suggests that the “optimal level” is not a static property of the stimulus but an emergent property of the interaction between the stimulus and the viewer’s interpretive efforts. A highly uncertain artwork can be just as pleasurable as a moderately uncertain one, provided that the viewer is equipped and motivated to engage in meaning-making. This reframes the debate from “what level of ambiguity is the best?” to “under what conditions can viewers successfully master ambiguity?”

Third, and most centrally, our work provides powerful empirical grounding for the Aesthetic of Reception theory (Iser, 1978; Jauss, 1982) and the “effort after meaning” principle (Bartlett, 1932). By demonstrating the superiority of active meaning-making over passive exposure or meaning-viewing (H1 and H2), we empirically validate the claim that the viewer’s role as a “co-creator” is the primary engine of aesthetic pleasure in the face of ambiguity. Our study moves this theory from the realm of literary criticism to testable psychological science.

To provide a clearer synthesis of how the meaning-driven uncertainty resolution model (MDURM) extends and diverges from these established frameworks, we systematically map its core components and predictions against these precursor theories in Table 4. This comparison highlights that while MDURM shares the drive for resolution with predictive processing and an emphasis on active agency with reception theory, it is distinct in regarding high uncertainty not as uniformly aversive but as a potential “gateway” to pleasure through specific, effortful meaning-making strategies. Crucially, while the act of engagement (process) is the vehicle for meaning-making, the aesthetic reward experience appears to be primarily contingent upon the subjective success of the resolution (outcome). This implies that active engagement is the most hedonically rewarding when it leads to a perceptible reduction in uncertainty, thereby satisfying the viewer’s need for certainty.

Beyond a component-level comparison, Table 4 also clarifies the theoretical status of the five hypotheses tested in the present research. Specifically, H1 (the superiority of active meaning-making) corresponds to the emphasis on active agency highlighted in reception theory and Bartlett’s “effort after meaning” but is not explicitly specified in fluency theory or Berlyne’s framework. H2 (uncertainty reduction as mediator) aligns conceptually with predictive-processing accounts, yet MDURM makes this mechanism explicit at the level of the aesthetic evaluation. Importantly, H3 (the strategy–uncertainty fit) and H4 (certainty-need satisfaction as a mediator) are not directly articulated in the precursor frameworks and, therefore, represent novel extensions introduced by the present model. Finally, H5 (the reduction of boredom through meaning-making) connects partially to arousal-based perspectives but is uniquely integrated into a strategy-dependent account in MDURM. In this way, the hypotheses tested herein both converge with and extend prior theories.

Our findings also revealed an unexpected nuance regarding mere exposure. Counterintuitively, the mere exposure condition in Study 1 yielded smaller increases in liking and beauty than the control condition. Although repetition often increases perceptual fluency and can enhance evaluations (Zajonc, 1968), our results suggest a potential boundary condition for complex artistic stimuli under rapid repetition. Specifically, our paradigm—involving brief, repeated presentations—may have induced affective habituation (Leventhal et al., 2007) and an “art fatigue” effect (Mikuni et al., 2022), which can emerge early and has been observed across cultural contexts (Mikuni et al., 2022; Xu & Zhao, 2023). From this perspective, repeated passive viewing may quickly reduce novelty or engagement for complex artworks, thereby counteracting any fluency-based benefits. By contrast, more sustained viewing (as in our control condition) and/or active interpretive engagement may help to maintain engagement and evaluative responses over repeated encounters. This observation, while secondary to our main hypotheses, highlights that the effects of passive exposure are not universally positive and depend critically on the interplay between the stimulus complexity and task parameters.

Beyond establishing the importance of meaning-making, our research dissects its internal mechanisms. The finding that the optimal strategy—intent based vs. self-associative—depends on the artwork’s uncertainty level (H3) offers an empirical solution to the art-theoretical debate on intentionality. It suggests that both “intentionalism” and “anti-intentionalism” capture valid aspects of the aesthetic experience. The viewer, as an adaptive cognitive agent, implicitly chooses the most efficient path to resolution: seeking external, authorial structure when it is likely present and generating internal, personal structure when it is not. This highlights the remarkable flexibility of our aesthetic-processing systems.

Furthermore, the robust mediating role of certainty-need satisfaction (H4) provides a crucial motivational anchor for these cognitive processes. As conceptualized in Study 3, the provision of multiple interpretations successfully enhanced certainty satisfaction, underscoring that in aesthetic contexts, certainty may be more about confirming a work’s interpretability than about finding a single truth. This finding is significant because it connects the rarified experience of art appreciation to a fundamental, domain-general psychological drive (Kruglanski & Webster, 1996). It suggests that engaging with challenging art is not merely a frivolous pastime but also can be a microcosm of how we cope with uncertainty in life: By actively imposing meaning and structure, we restore a sense of cognitive order and competence, which is intrinsically rewarding. This also explains the powerful role of meaning-making in alleviating boredom (H5), which can be conceptualized as a “crisis of meaning” (Danckert & Elpidorou, 2023). The present work documents robust boredom reductions under meaning-making; unpacking whether this effect operates via increased perceived meaning remains an important next step.

### 5.2. Practical Implications

Our findings have significant practical implications. For art education, our results suggest that instead of simply providing students with canonical interpretations, educators should foster skills for active, personal meaning-making to enhance appreciation for complex art. For museums and galleries, curators could design interactive exhibits that prompt viewers to articulate their interpretations, thereby deepening engagement and enjoyment. The principles uncovered herein may also inform therapeutic practices, using engagement with art as a tool to combat boredom and cultivate a sense of agency and meaning.

At the same time, these recommendations warrant a note of caution. Active engagement—such as inferring artist intention or reflecting on self-related meaning—can be cognitively demanding, particularly for novices, and sustained effort across many works may become taxing over time. Thus, guided meaning-making may be the most effective when implemented with appropriate “dosages” and scaffolding—for example, focusing deeply on a small number of artworks (e.g., a seminar-style discussion of a single painting or a museum station designed for extended reflection) rather than requiring intensive interpretation for every work in a long sequence. In settings involving many artworks (e.g., gallery tours or courses), lighter-touch prompts (e.g., brief interpretive questions, optional reflection moments, or alternating between active and passive viewing) may help to prevent fatigue and maintain engagement.

Future research could further refine these boundary conditions by systematically varying the number of artworks, viewing pace, and level of interpretive guidance and by testing moderating factors, such as art expertise, motivation, and cognitive load. Such work would help to specify when and for whom active engagement interventions maximize benefits without becoming counterproductive.

### 5.3. Limitations and Future Directions

While our findings provide a coherent narrative, several limitations highlight avenues for future research. First, our experimental design, which instructed participants on which strategy to use, allowed for controlled causal inference but precluded the observation of spontaneously chosen strategies. Future work could employ more naturalistic paradigms to investigate what factors (e.g., personality traits, like the need for cognitive closure or art expertise) predict which meaning-making path an individual will spontaneously take.

Second, in Study 1, the exposure duration differed across experimental conditions. The elaborative tasks (making meaning, viewing meaning, and the control) allowed up to three minutes of engagement, whereas the mere exposure condition consisted of brief repeated presentations. Although this difference was theoretically embedded in the intended manipulation of the cognitive engagement—contrasting sustained semantic elaboration with rapid perceptual processing—it remains possible that the viewing time contributed to the observed effects. Future research could further disentangle the exposure duration and engagement processes by equating the viewing time more strictly across conditions while preserving the intended cognitive manipulations.

Third, our focus on visual art raises questions of modality generality. While our model aligns with findings in music perception, the specific interplay of intentionality and self-relevance might differ across art forms. The author is arguably more salient in literature than in abstract painting or instrumental music, suggesting the dynamics we uncovered may be modality specific.

Fourth, the construct of “certainty-need satisfaction” itself warrants deeper investigation. Is the pleasure derived from achieving a single, stable interpretation or from the confidence that an interpretation is possible, even if multiple interpretations exist? Our Study 3 hints at the latter, but future research could directly contrast these forms of “certainty” to further refine our understanding of the epistemic rewards of art.

Fifth, the generalizability of our findings is constrained by the characteristics of our sample. Our participants were primarily young, university-educated Chinese adults. This demographic homogeneity warrants caution when extending our conclusions to the broader population. For instance, age and accumulated life experience are likely to be crucial moderators of the meaning-making process. Older adults, possessing a richer repository of personal memories and world knowledge, might engage in self-associative meaning-making more readily or with greater depth, which could potentially alter the relative effectiveness of the strategies we examined.

Similarly, the cultural background of our participants is a critical contextual factor. Our findings, derived from Chinese individuals viewing Western art, may not directly generalize to Western audiences or to the appreciation of non-Western art traditions, where aesthetic norms and interpretive styles may differ. Therefore, future research is essential to test the robustness of our model across diverse age groups, gender distributions, educational backgrounds, and cultural contexts. Replicating these findings with more demographically representative samples is a crucial next step in establishing the universality of the meaning-driven uncertainty resolution model.

## Figures and Tables

**Table 1:** Fixed effects from the linear mixed models constructed to examine the consequences of picture types (PTs) and uncertainty reduction modes (URMs) for ratings of uncertainty reduction, increased liking, increased beauty, boredom reduction, and increased pleasure.

Fixed Effect	*b*	*SE*	*t*-Value	*p*-Value
**Uncertainty Reduction**				
Intercept	0.12	0.09	1.28	0.201
PT (moderate uncertainty)	0.71	0.13	5.36	<0.001
PT (high uncertainty)	1.55	0.13	11.89	<0.001
URM (viewing meaning)	0.13	0.13	0.94	0.349
URM (mere exposure)	0.13	0.13	0.96	0.338
URM (control)	−0.13	0.13	−0.99	0.322
PT (moderate uncertainty): URM (viewing meaning)	−0.34	0.19	−1.83	0.068
PT (high uncertainty): URM (viewing meaning)	−1.13	0.19	−5.97	<0.001
PT (moderate uncertainty): URM (mere exposure)	−0.55	0.19	−2.89	0.004
PT (high uncertainty): URM (mere exposure)	−1.38	0.18	−7.65	<0.001
PT (moderate uncertainty): URM (control)	−0.60	0.18	−3.23	0.001
PT (high uncertainty): URM (control)	−1.04	0.18	−5.61	<0.001
Conditional *R*^2^		0.34		
Marginal *R*^2^		0.14		
**Increased Liking**				
Intercept	0.85	0.07	12.09	<0.001
PT (moderate uncertainty)	0.32	0.10	3.14	0.002
PT (high uncertainty)	0.56	0.10	5.62	<0.001
URM (viewing meaning)	−0.50	0.10	−4.89	<0.001
URM (mere exposure)	−0.88	0.10	−8.72	<0.001
URM (control)	−0.76	0.10	−7.59	<0.001
PT (moderate uncertainty): URM (viewing meaning)	0.11	0.14	0.78	0.435
PT (high uncertainty): URM (viewing meaning)	−0.14	0.14	−0.96	0.337
PT (moderate uncertainty): URM (mere exposure)	−0.12	0.15	−0.85	0.396
PT (high uncertainty): URM (mere exposure)	−0.21	0.14	−1.54	0.124
PT (moderate uncertainty): URM (control)	−0.18	0.14	−1.30	0.192
PT (high uncertainty): URM (control)	−0.23	0.14	−1.64	0.102
Conditional *R*^2^	0.32			
Marginal *R*^2^	0.19			
**Increased Beauty**				
Intercept	0.82	0.07	11.84	<0.001
PT (moderate uncertainty)	0.27	0.10	2.73	0.007
PT (high uncertainty)	0.52	0.10	5.28	<0.001
URM (viewing meaning)	−0.50	0.10	−5.17	<0.001
URM (mere exposure)	−0.74	0.10	−7.78	<0.001
URM (control)	−0.69	0.09	−7.42	<0.001
PT (moderate uncertainty): URM (viewing meaning)	0.16	0.14	1.18	0.234
PT (high uncertainty): URM (viewing meaning)	−0.07	0.14	−0.53	0.599
PT (moderate uncertainty): URM (mere exposure)	−0.25	0.14	−1.82	0.069
PT (high uncertainty): URM (mere exposure)	−0.24	0.13	−1.84	0.066
PT (moderate uncertainty): URM (control)	−0.15	0.13	−1.14	0.256
PT (high uncertainty): URM (control)	−0.33	0.13	−2.51	0.013
Conditional *R*^2^		0.34		
Marginal *R*^2^		0.19		
**Boredom Reduction**				
Intercept	0.32	0.09	3.58	<0.001
PT (moderate uncertainty)	0.21	0.13	1.62	0.106
PT (high uncertainty)	0.97	0.13	7.53	<0.001
URM (viewing meaning)	0.01	0.13	0.07	0.943
URM (mere exposure)	−0.23	0.13	−1.80	0.071
URM (control)	−0.28	0.13	−2.20	0.028
PT (moderate uncertainty): URM (viewing meaning)	0.08	0.18	0.44	0.658
PT (high uncertainty): URM (viewing meaning)	−0.59	0.19	−3.16	0.001
PT (moderate uncertainty): URM (mere exposure)	0.14	0.19	0.76	0.445
PT (high uncertainty): URM (mere exposure)	−0.66	0.18	−3.72	<0.001
PT (moderate uncertainty): URM (control)	−0.30	0.18	−1.65	0.099
PT (high uncertainty): URM (control)	−0.64	0.18	−3.54	<0.001
Conditional *R*^2^		0.26		
Marginal *R*^2^		0.08		
**Increased Pleasure**				
Intercept	0.81	0.07	11.74	<0.001
PT (moderate uncertainty)	0.39	0.10	4.02	<0.001
PT (high uncertainty)	0.48	0.10	4.94	<0.001
URM (viewing meaning)	−0.48	0.10	−4.72	<0.001
URM (mere exposure)	−0.76	0.10	−7.62	<0.001
URM (control)	−0.75	0.10	−7.75	<0.001
PT (moderate uncertainty): URM (viewing meaning)	0.03	0.14	0.18	0.86
PT (high uncertainty): URM (viewing meaning)	0.02	0.14	0.13	0.90
PT (moderate uncertainty): URM (mere exposure)	−0.13	0.14	−0.91	0.37
PT (high uncertainty): URM (mere exposure)	−0.18	0.14	−1.34	0.18
PT (moderate uncertainty): URM (control)	−0.23	0.14	−1.70	0.09
PT (high uncertainty): URM (control)	−0.14	0.14	−1.03	0.30
Conditional *R*^2^		0.26		
Marginal *R*^2^		0.15		

Note. In all the LMMs, the reference level for the uncertainty reduction mode (URM) is “making meaning”; coefficients, therefore, represent contrasts vs. “making meaning”. Pairwise comparisons between all the conditions (Tukey adjusted) are reported in the main text.

**Table 2:** Fixed effects from the linear mixed models constructed to examine the consequences of picture types (PTs) and meaning-making types (MMTs) for ratings of uncertainty reduction, increased liking, increased beauty, boredom reduction, and increased pleasure.

Fixed Effect	*b*	*SE*	*t* Value	*p* Value
**Uncertainty Reduction**				
Intercept	0.39	0.12	3.16	0.002
PT (moderate uncertainty)	0.77	0.17	4.47	<0.001
PT (high uncertainty)	0.81	0.17	4.46	<0.001
MMT (self-association)	−0.29	0.16	−1.83	0.069
PT (moderate uncertainty): MMT (self-association)	−0.33	0.22	−1.52	0.129
PT (high uncertainty): MMT (self-association)	0.71	0.22	3.24	0.001
Conditional *R*^2^			0.32	
Marginal *R*^2^			0.15	
**Increased Liking**				
Intercept	1.21	0.12	9.80	<0.001
PT (moderate uncertainty)	0.77	0.17	4.56	<0.001
PT (high uncertainty)	−0.67	0.17	−3.98	<0.001
MMT (self-association)	−0.60	0.17	−3.51	<0.001
PT (moderate uncertainty): MMT (self-association)	−0.65	0.23	−2.76	0.006
PT (high uncertainty): MMT (self-association)	1.59	0.23	6.80	<0.001
Conditional *R*^2^			0.39	
Marginal *R*^2^			0.16	
**Increased Beauty**				
Intercept	1.17	0.13	9.14	<0.001
PT (moderate uncertainty)	0.71	0.18	4.01	<0.001
PT (high uncertainty)	−0.67	0.17	−3.86	<0.001
MMT (self-association)	−0.59	0.18	−3.30	0.001
PT (moderate uncertainty): MMT (self-association)	−0.64	0.24	−2.63	0.009
PT (high uncertainty): MMT (self-association)	1.58	0.24	6.50	<0.001
Conditional *R*^2^			0.52	
Marginal *R*^2^			0.18	
**Boredom Reduction**				
Intercept	−0.30	0.13	−2.45	0.015
PT (moderate uncertainty)	−0.60	0.17	−3.57	<0.001
PT (high uncertainty)	−0.31	0.17	−1.84	0.067
MMT (self-association)	0.35	0.17	2.15	0.033
PT (moderate uncertainty): MMT (self-association)	0.00	0.23	0.02	0.986
PT (high uncertainty): MMT (self-association)	−1.09	0.23	−4.82	<0.001
Conditional *R*^2^			0.28	
Marginal *R*^2^			0.10	
**Increased Pleasure**				
Intercept	−0.30	0.12	−2.45	0.015
PT (moderate uncertainty)	−0.60	0.17	−3.57	<0.001
PT (high uncertainty)	−0.31	0.17	−1.84	0.067
MMT (self-association)	0.35	0.16	2.15	0.033
PT (moderate uncertainty): MMT (self-association)	0.00	0.23	0.02	0.986
PT (high uncertainty): MMT (self-association)	−1.09	0.23	−4.82	<0.001
Conditional *R*^2^			0.45	
Marginal *R*^2^			0.14	

**Table 3:** Fixed effects from the linear mixed models constructed to examine the consequences of picture types (PTs) and the number of meanings (NMs) for ratings of increased liking, increased beauty, boredom reduction, and increased pleasure.

Fixed Effect	*b*	*SE*	*t* Value	*p* Value
**Increased Liking**				
Intercept	0.43	0.03	16.37	<0.001
PT (moderate uncertainty)	0.56	0.04	15.16	<0.001
PT (high uncertainty)	0.56	0.04	15.09	<0.001
NM (three meanings)	0.17	0.04	4.48	<0.001
NM (five meanings)	0.85	0.04	22.75	<0.001
PT (moderate uncertainty): NM (three meanings)	0.50	0.05	9.51	<0.001
PT (high uncertainty): NM (three meanings)	0.25	0.05	4.84	<0.001
PT (moderate uncertainty): NM (five meanings)	0.16	0.05	2.98	0.003
PT (high uncertainty): NM (five meanings)	0.11	0.05	2.17	0.031
Conditional *R*^2^			0.74	
Marginal *R*^2^			0.70	
**Increased Beauty**				
Intercept	0.36	0.04	9.67	<0.001
PT (moderate uncertainty)	0.61	0.05	11.57	<0.001
PT (high uncertainty)	0.70	0.05	13.28	<0.001
NM (three meanings)	0.23	0.04	5.26	<0.001
NM (five meanings)	0.97	0.04	22.29	<0.001
PT (moderate uncertainty): NM (three meanings)	0.41	0.06	6.73	<0.001
PT (high uncertainty): NM (three meanings)	0.13	0.06	2.05	0.041
PT (moderate uncertainty): NM (five meanings)	0.02	0.06	0.34	0.734
PT (high uncertainty): NM (five meanings)	−0.12	0.06	−1.95	0.052
Conditional *R*^2^			0.70	
Marginal *R*^2^			0.64	
**Boredom Reduction**				
Intercept	0.40	0.04	10.25	<0.001
PT (moderate uncertainty)	0.40	0.06	7.20	<0.001
PT (high uncertainty)	0.60	0.06	10.75	<0.001
NM (three meanings)	0.29	0.05	5.41	<0.001
NM (five meanings)	0.48	0.05	8.99	<0.001
PT (moderate uncertainty): NM (three meanings)	0.27	0.08	3.66	<0.001
PT (high uncertainty): NM (three meanings)	0.18	0.07	2.37	0.018
PT (moderate uncertainty): NM (five meanings)	0.46	0.08	6.11	<0.001
PT (high uncertainty): NM (five meanings)	0.41	0.08	5.43	<0.001
Conditional *R*^2^			0.52	
Marginal *R*^2^			0.46	
**Increased Pleasure**				
Intercept	0.36	0.03	12.41	<0.001
PT (moderate uncertainty)	0.61	0.04	14.76	<0.001
PT (high uncertainty)	0.69	0.04	16.55	<0.001
NM (three meanings)	0.21	0.04	5.04	<0.001
NM (five meanings)	0.82	0.04	19.66	<0.001
PT (moderate uncertainty): NM (three meanings)	0.45	0.06	7.63	<0.001
PT (high uncertainty): NM (three meanings)	0.10	0.06	1.73	0.084
PT (moderate uncertainty): NM (five meanings)	0.21	0.06	3.57	<0.001
PT (high uncertainty): NM (five meanings)	0.10	0.06	1.73	0.084
Conditional *R*^2^			0.69	
Marginal *R*^2^			0.65	

**Table 4:** Systematic comparison of the meaning-driven uncertainty resolution model (MDURM) with precursor theories.

Theoretical Component/Prediction	Processing Fluency Theory	Predictive Processing	Berlyne’s Arousal Theory	Bartlett’s “Effort after Meaning”	Iser’s Aesthetic of Reception	MDURM (Current Model)
**1. Role of high uncertainty/ambiguity**	− (aversive; implies low fluency)	+ (prediction error as a signal to be resolved)	− (aversive; linked to over-arousal)	+ (triggers cognitive effort)	+ (indeterminacy as a structural feature)	+ (a potential “gateway” under active engagement)
**2. Primary source of aesthetic pleasure**	+ (processing ease/fluency)	+ (successful reduction of prediction error)	+ (optimal arousal level)	+ (effortful meaning construction)	+ (“concretization” of meaning)	+ (active uncertainty resolution and certainty-need satisfaction)
**3. Passive vs. active processing emphasis**	? (often implicit/automatic)	? (can be automatic or active, depending on the context)	? (mostly stimulus driven)	+ (active effort is central)	+ (viewer as co-creator)	+ (active agency is central)
**4. Inverted-U relation between uncertainty and pleasure**	? (typically, a linear fluency–preference relation)	? (focuses on dynamics of error reduction rather than fixed levels)	+ (core theoretical assumption)	? (process oriented not stimulus level)	? (structure oriented)	± (preference depends on strategy and engagement mode)
**5. Role of meaning-making strategies**	? (not a core construct)	+ (model updating and sense-making)	? (exploration without explicit semantics)	+ (schema construction)	+ (gap-filling)	+ (strategy dependent: intent based vs. self-associative)
**6. Explicit motivational mechanism**	? (hedonic marking of fluency)	+ (epistemic motivation/order)	+ (arousal regulation)	+ (drive to comprehend)	? (implicit reader involvement)	+ (the need for certainty as an explicit mediator)
**7. Role of boredom**	? (not a central construct)	+ (perfect predictability implies low engagement)	+ (low arousal linked to boredom)	? (not explicitly addressed)	? (not explicitly addressed)	+ (meaning-making reduces boredom; H5)

Notes: “+” indicates a component is explicitly emphasized; “-” indicates an opposing view or a negative relationship predicted by the theory; “?” indicates that the component is not central or is underspecified; “±” indicates a context-dependent or interaction-based prediction. The table provides a conceptual comparison rather than an exhaustive evaluation of each framework.

## Data Availability

The materials and data are available at https://osf.io/csd7q/?view_only=a01a74a5a9a240c9876fd76ae45148c4; accessed on 7 May 2025.

## Supplementary Materials

The following supporting information can be downloaded at https://www.mdpi.com/article/10.3390/bs16020286/s1: Tables S1–S5: Detailed Stimulus Metadata, Empirical Pretest Ratings, and Descriptive Statistics for Studies 1–3.
