# Supplementary material for: Uncertainty as a Gateway to Beauty: The Impact of Uncertainty Reduction on Art Appreciation
Source: Behav Sci (Basel). 2026 Feb 16;16(2):286. doi: 10.3390/bs16020286 (PMC12938020; doi:10.3390/bs16020286)
Supplement: Supplementary file 1 [file behavsci-16-00286-s001.zip › behavsci-4043197-supplementary.pdf]

Supplementary Materials

Uncertainty as a Gateway to Beauty: The Impact of Uncertainty Reduction on Art Appreciation

Table of Contents

Table S1. Detailed Metadata for the 24 Experimental Stimuli (Paintings).

Table S2. Empirical Ratings (Uncertainty, Beauty, Liking, Familiarity, Valence) for the Initial Set of 54 Paintings

Table S3. Means and Standard Deviations for uncertainty reduction, increased liking, increased beauty, boredom reduction and increased pleasure Ratings Grouped by Picture Types and Uncertainty Reduction Modes (Study 1).

Table S4. Means and Standard Deviations for uncertainty reduction, increased liking, increased beauty, boredom reduction and increased pleasure Ratings Grouped by Picture Types and Meaning-Making Types (Study 2).

Table S5. Means and Standard Deviations for uncertainty reduction, increased liking, increased beauty, boredom reduction and increased pleasure Ratings Grouped by Picture Types and Meaning-Making Types (Study 3).

Table S1 Detailed Metadata for the 24 Experimental Stimuli (Paintings).

Image ID	Artist	Title	Date	Style / Movement	Source	Pixel
		**Low Uncertainty Stimuli (d-series)**				
d1	Cynthia Feustel	*Kodi*	Contemporary	Contemporary Realism	https://www.artworkarchive.com/profile/cynthia-feustel/artwork/kodi	635 x 847
d2	Pedro Campos	*A Hot Day*	2000s	Hyperrealism	https://www.plusonegallery.com/artworks/1560/	848 x 847
d3	Suzanne Eisendieck	*A Beautiful Sunday*	c. 1960	Impressionism	https://www.1stdibs.com/art/paintings/figurative-paintings/suzanne-eisendieck-beautiful-sunday-beau-dimanche-oil-painting-impressionist-style/id-a_173182/	1029 x 847
d4	Marcel Dyf	*Girl in Green Corsage*	20th C.	Post-Impressionism	https://wahooart.com/sk/art/marcel-dyf-girl-in-green-corsage-A26BZD-en/	683 x 847
d5	Marcel Dyf	*Peonies and Anemones*	20th C.	Impressionism	https://gallerix.org/storeroom/622572445/N/1579711209/	691 x 847
d6	Wu Feng (吴峰)	*Still Life with Persimmons*	2014	Contemporary Realism	https://huaban.com/pins/3953108192	670 x 847

d7	Wu Feng (吴峰)	*Narcissus*	2015	Contemporary Realism	http://www.360doc.com/content/17/0327/14/40833005_640543803.shtml	656 x 847
d8	Andrew Wyeth	*Distant Thunder*	1961	Regionalism / Realism	https://www.artsy.net/artwork/andrew-wyeth-distant-thunder	519 x 847
		**Moderate Uncertainty Stimuli (m-series)**				
m1	Dan McCaw	*The Diner*	Contemporary	American Impressionism	https://www.tuttartpitturasculturapoesiamusica.com/2011/12/dan-mccaw-1942-american-expressionist.html	640 x 847
m2	Ian Houston	*Street Market in the Western District*	20th C.	Contemporary Impressionism	https://www.panterandhall.com/products/street-market-in-the-western-district-hong-kong	1457 x 847
m3	Vasily Kandinsky	*Two on a horse*	1906–07	Expressionism	https://gallerix.asia/storeroom/1193097145/N/1267394244/	757 x 847
m4	Ian Houston	*Landscape (Norfolk)*	20th C.	Contemporary Impressionism	https://www.panterandhall.com/products/ian-houston-passing-holkham	1012 x 847
m5	René Magritte	*The Infinite Recognition*	1963	Surrealism	https://www.wikiart.org/en/rene-magritte/the-infinite-recognition-1963	1023 x 847

m6	Louis Abel-Truchet	*In the Orchard*	c. 1913	Symbolism / Realism	https://www.1stdibs.com/art/paintings/landscape-paintings/louis-abel-truchet-orchard-louis-abel-truchet-1857-1918/id-a_1045813/	688 x 847
m7	Louis Abel-Truchet	*La Fete Forain*	c. 1900	Post-Impressionism	https://fineartamerica.com/featured/la-fete-forain-louis-abel-truchet.html	857 x 847
m8	Marc Chagall	*The Promenade*	1917–18	Surrealism / Cubism	https://www.amazon.se/-/en/Chagall-Picture-Canvas-Promenad-Painting/dp/B0B3DN5PTT	843 x 847
			**High Uncertainty Stimuli (m-series)**			
g1	Joan Miró	*The Hunter (Catalan Landscape)*	1923–24	Surrealism	https://www.artera.ae/artworks/f5d7b297-f69e-4719-9387-3742d1e5b0f7	1334 x 847
g2	Barnett Newman	*Be I*	1970	Abstract Expressionism (Color Field)	https://www.artera.ae/artworks/2ac8d180-0efb-4684-9670-c6946ca94a64	525 x 847
g3	Wassily Kandinsky	*Composition IV*	1911	Abstract Art	https://arthive.com/zh/wasilykandinsky/works/212270~Composition_4	1339 x 847

g4	Marc Chagall	*I and the Village*	1911	Cubism / Modernism	https://www.marcchagall.net/i-and-the-village.jsp	505 x 847
g5	Demeter	*The Dream*	Modern	Line Art / Minimalism	http://www.etoff.com.cn/html/qiyizhan/430.html	1750 x 847
g6	Marc Chagall	*Paradise*	1961	Modernism / Biblical Art	https://www.wikiart.org/en/marc-chagall/paradise-1961-7	848 x 847
g7	Marc Chagall	*Le grand soleil*	1978	Surrealism / Modernism	https://www.mutualart.com/Artwork/Le-grand-soleil/1E88813F536CE49E	1289 x 847
g8	Marc Chagall	*The Drunkard*	1911–12	Cubism / Fauvism	https://en.wikipedia.org/wiki/File:Marc_Chagall,_1911-12,_The_Drunkard_(Le_saoul),_1912,_oil_on_canvas._85_x_115_cm._Private_collection.jpg	737 x 847

Table S2. Empirical Ratings (Uncertainty, Beauty, Liking, Familiarity, Valence) for the Initial Set of 54 Paintings

ID	Uncertainty M(SD)	Beauty M(SD)	Liking M(SD)	Familiarity M(SD)	Valence M(SD)
**Low Uncertainty (d-series)**					
d1	1.71 (0.82)	5.16 (1.21)	4.65 (1.58)	3.29 (1.77)	5.23 (1.02)
d2	1.74 (1.24)	5.23 (1.02)	5.26 (1.41)	3.97 (1.96)	5.68 (0.98)
d3	1.87 (1.34)	4.10 (1.47)	4.00 (1.48)	4.90 (2.21)	4.87 (1.18)
d4	1.87 (1.09)	4.55 (1.26)	4.23 (1.63)	5.00 (1.48)	4.55 (1.09)
d5	2.00 (1.00)	5.65 (1.20)	5.52 (1.46)	4.26 (1.83)	6.13 (0.88)
d6	2.13 (1.15)	5.16 (1.16)	4.84 (1.34)	4.16 (1.93)	5.32 (1.01)
d7	2.39 (1.43)	4.68 (1.08)	4.03 (1.58)	3.90 (1.49)	4.55 (1.09)
d8	2.39 (1.38)	4.71 (1.16)	4.42 (1.39)	4.55 (1.71)	5.13 (0.85)
**Moderate Uncertainty (m-series)**					
m1	3.23 (1.41)	5.74 (1.06)	5.48 (1.26)	3.94 (1.61)	5.29 (1.10)
m2	3.64 (1.29)	3.67 (1.26)	3.22 (1.61)	3.61 (1.50)	3.86 (1.38)
m3	3.81 (1.62)	4.17 (1.61)	3.75 (1.59)	3.58 (1.36)	4.92 (1.13)
m4	3.85 (1.33)	4.45 (1.20)	3.85 (1.60)	3.73 (1.55)	4.33 (0.99)
m5	4.03 (1.57)	4.30 (1.33)	3.85 (1.48)	3.67 (1.55)	4.24 (1.15)
m6	4.06 (1.64)	4.52 (1.35)	4.15 (1.70)	3.42 (1.58)	4.73 (1.42)
m7	4.28 (1.50)	4.31 (1.24)	3.89 (1.43)	3.39 (1.68)	4.33 (1.04)
m8	2.87 (1.34)	4.10 (1.47)	4.00 (1.48)	4.90 (2.21)	4.87 (1.18)
**High Uncertainty (g-series)**					
g1	5.61 (1.32)	4.08 (1.30)	3.72 (1.52)	2.47 (1.32)	4.33 (1.17)
g2	5.64 (1.19)	3.42 (1.48)	3.21 (1.63)	2.94 (1.48)	4.18 (0.95)
g3	5.94 (1.20)	3.73 (1.59)	3.36 (1.76)	2.45 (1.30)	4.03 (1.21)
g4	5.97 (1.55)	3.15 (1.54)	2.55 (1.25)	2.94 (1.90)	3.79 (1.08)
g5	6.00 (1.12)	3.61 (1.43)	3.24 (1.77)	2.55 (1.56)	3.85 (1.35)
g6	6.17 (1.23)	3.83 (1.38)	3.64 (1.51)	2.58 (1.66)	4.31 (0.95)
g7	6.33 (1.05)	2.67 (1.57)	2.27 (1.35)	2.55 (1.37)	2.67 (1.38)
g8	6.52 (0.80)	2.52 (1.33)	2.12 (1.43)	2.48 (1.84)	2.79 (1.45)

Table S3 Means and Standard Deviations for uncertainty reduction, increased liking, increased beauty, boredom reduction and increased pleasure Ratings Grouped by Picture Types and Uncertainty Reduction Modes (Study 1).

	Low uncertainty	Moderate uncertainty	High uncertainty	Overall
**Uncertainty Reduction**				
Making meaning	0.12 (0.97)	0.83 (1.09)	1.67 (1.23)	0.86 (1.27)
Viewing meaning	0.25 (0.62)	0.62 (0.39)	0.67 (0.81)	0.52 (0.65)
Mere exposure	0.25 (1.18)	0.41 (1.50)	0.41 (1.01)	0.36 (1.21)
Control group	-0.01 (1.35)	0.11 (1.03)	0.50 (1.28)	0.20 (1.25)
Overall	0.15 (1.07)	0.50 (1.09)	0.80 (1.20)	
**Increased Liking**				
Making meaning	0.85 (0.74)	1.16 (0.96)	1.41 (1.08)	1.13 (0.96)
Viewing meaning	0.34 (0.34)	0.77 (0.43)	0.77 (0.51)	0.63 (0.47)
Mere exposure	-0.04 (1.10)	0.15 (1.39)	0.31 (1.10)	0.16 (1.19)
Control group	0.09 (0.80)	0.22 (0.72)	0.42 (1.07)	0.24 (0.88)
Overall	0.33 (0.87)	0.60 (1.01)	0.70 (1.08)	
**Increased Beauty**				
Making meaning	0.82 (0.72)	1.09 (0.92)	1.34 (1.02)	1.08 (0.92)
Viewing meaning	0.32 (0.40)	0.75 (0.45)	0.77 (0.59)	0.62 (0.53)
Mere exposure	0.08 (0.94)	0.09 (1.21)	0.36 (0.97)	0.20 (1.04)
Control group	0.13 (0.72)	0.24 (0.68)	0.32 (0.99)	0.23 (0.81)
Overall	0.35 (0.78)	0.57 (0.93)	0.68 (1.00)	
**Boredom Reduction**				
Making meaning	0.32 (0.77)	0.53 (1.01)	1.29 (1.53)	0.71 (1.21)
Viewing meaning	0.33 (0.41)	0.62 (0.45)	0.72 (0.75)	0.56 (0.58)
Mere exposure	0.09 (1.16)	0.44 (1.52)	0.40 (1.51)	0.32 (1.42)
Control group	0.04 (1.08)	-0.05 (0.96)	0.37 (1.46)	0.12 (1.20)
Overall	0.20 (0.92)	0.38 (1.06)	0.68 (1.42)	
**Increased Pleasure**				
Making meaning	0.81 (0.85)	1.20 (0.98)	1.29 (1.15)	1.09 (1.02)
Viewing meaning	0.33 (0.38)	0.75 (0.44)	0.83 (0.49)	0.65 (0.49)
Mere exposure	0.05 (1.34)	0.31 (1.28)	0.35 (1.03)	0.25 (1.21)
Control group	0.05 (0.98)	0.21 (0.85)	0.39 (1.10)	0.22 (0.99)
Overall	0.33 (1.00)	0.64 (1.00)	0.70 (1.06)	

Table S4 Means and Standard Deviations for uncertainty reduction, increased liking, increased beauty, boredom reduction and increased pleasure Ratings Grouped by Picture Types and Meaning-Making Types (Study 2).

	Low uncertainty	Moderate uncertainty	High uncertainty	Overall
**Uncertainty Reduction**				
Based on author's intention	0.39 (1.48)	1.16 (1.17)	1.20 (1.48)	0.94 (1.43)
Self-association	0.10 (0.86)	0.54 (1.25)	1.62 (1.21)	0.77 (1.29)
Overall	0.24 (1.21)	0.83 (1.25)	1.41 (1.36)	
**Increased Liking**				
Based on author's intention	1.21 (1.14)	1.98 (1.17)	0.54 (1.49)	1.23 (1.42)
Self-association	0.61 (0.93)	0.74 (1.24)	1.53 (1.12)	0.97 (1.18)
Overall	0.90 (1.08)	1.32 (1.36)	1.04 (1.41)	
**Increased Beauty**				
Based on author's intention	1.17 (1.05)	1.88 (1.17)	0.50 (1.22)	1.17 (1.29)
Self-association	0.58 (0.77)	0.65 (1.09)	1.49 (1.10)	0.92 (1.09)
Overall	0.87 (0.96)	1.23 (1.28)	1.00 (1.26)	
**Boredom Reduction**				
Based on author's intention	0.30 (1.17)	0.91 (1.10)	0.61 (1.64)	
Self-association	-0.05 (0.90)	0.55 (1.31)	1.34 (1.49)	
Overall	0.12 (1.05)	0.72 (1.23)	0.98 (1.60)	
**Increased Pleasure**				
Based on author's intention	1.02 (1.38)	1.83 (1.33)	0.50 (1.28)	1.11 (1.44)
Self-association	0.54 (0.92)	0.69 (1.12)	1.41 (1.20)	0.89 (1.15)
Overall	0.77 (1.19)	1.23 (1.35)	0.96 (1.32)	

Table S5 Means and Standard Deviations for uncertainty reduction, increased liking, increased beauty, boredom reduction and increased pleasure Ratings Grouped by Picture Types and Meaning-Making Types (Study 3).

	Low uncertainty	Moderate uncertainty	High uncertainty	Overall
**Increased Liking**				
1-meanings	0.43 (0.43)	0.99 (0.21)	0.99 (0.36)	0.80 (0.43)
3-meanings	0.60 (0.22)	1.66 (0.30)	1.41 (0.35)	1.23 (0.54)
5-meanings	1.28 (0.45)	2.00 (0.34)	1.95 (0.37)	1.75 (0.51)
Overall	0.77 (0.53)	1.55 (0.51)	1.45 (0.53)	
**Increased Beauty**				
1-meanings	0.36 (0.40)	0.97 (0.27)	1.06 (0.54)	0.79 (0.52)
3-meanings	0.59 (0.17)	1.61 (0.42)	1.41 (0.31)	1.21 (0.54)
5-meanings	1.32 (0.36)	1.95 (0.46)	1.90 (0.45)	1.73 (0.51)
Overall	0.75 (0.53)	1.51 (0.56)	1.46 (0.56)	
**Boredom Reduction**				
1-meanings	0.40 (0.47)	0.80 (0.30)	1.00 (0.69)	0.73 (0.57)
3-meanings	0.69 (0.30)	1.36 (0.33)	1.46 (0.60)	1.18 (0.55)
5-meanings	0.88 (0.58)	1.74 (0.61)	1.88 (0.57)	1.51 (0.73)
Overall	0.66 (0.50)	1.30 (0.58)	1.45 (0.72)	
**Increased Pleasure**				
1-meanings	0.36 (0.45)	0.98 (0.26)	1.05 (0.31)	0.80 (0.46)
3-meanings	0.57 (0.14)	1.64 (0.30)	1.36 (0.40)	1.20 (0.54)
5-meanings	1.18 (0.79)	2.01 (0.32)	1.97 (0.24)	1.73 (0.63)
Overall	0.70 (0.63)	1.54 (0.52)	1.46 (0.50)	
